# Phylogenetic Characterization of Arboviruses in Patients Suffering from Acute Fever in Rondônia, Brazil

**DOI:** 10.3390/v12080889

**Published:** 2020-08-14

**Authors:** Jackson Alves da Silva Queiroz, Luan Felipo Botelho-Souza, Felipe Souza Nogueira-Lima, Rita de Cássia Pontello Rampazzo, Marco Aurélio Krieger, Miriam Ribas Zambenedetti, Fabricio Klerinton Marchini, Ivo Alberto Borghetti, Dhelio Batista Pereira, Juan Miguel Vilalobos Salcedo, Deusilene Souza Vieira, Alcione de Oliveira dos Santos

**Affiliations:** 1 Oswaldo Cruz Foundation of Rondônia—FIOCRUZ/RO, Porto Velho RO 76812 245, Rondônia, Brazil; queiroz.jas@gmail.com (J.A.d.S.Q.); luan_botelho@hotmail.com (L.F.B.-S.); limafsn@hotmail.com (F.S.N.-L.); juanitto2001@yahoo.com.br (J.M.V.S.); deusylenebio@hotmail.com (D.S.V.); 2 Postgraduate Program in Experimental Biology of Federal University of Rondônia—PGBIOEXP, Porto Velho RO 76801 059, Rondônia, Brazil; 3 National Institute of Epidemiology of Western Amazonia—INCT EpiAmO, Porto Velho RO 76812 245, Rondônia, Brazil; 4 Aparício Carvalho University Center, Porto Velho RO 76811-678, Rondônia, Brazil; 5 Institute of Molecular Biology of Paraná -IBMP, Curitiba PR 81350-010, Rondônia, Brazil; rcprampazzo@gmail.com (R.d.C.P.R.); marco.krieger@fiocruz.br (M.A.K.); miriamrzam@gmail.com (M.R.Z.); markinifk@ibmp.org.br (F.K.M.); ivoborghetti@gmail.com (I.A.B.); 6 Tropical Medicine of Rondônia Center Research—CEPEM/RO, Porto Velho RO 76812 329, Rondônia, Brazil; dbpfall@gmail.com

**Keywords:** arboviruses, phylogenetic, genotyping

## Abstract

The purpose of the study was to classify, through phylogenetic analyses, the main arboviruses that have been isolated in the metropolitan region of Porto Velho, Rondônia, Brazil. Serum samples from patients with symptoms suggesting arboviruses were collected and tested by One Step RT-qPCR for Zika, Dengue (serotypes 1–4), Chikungunya, Mayaro and Oropouche viruses. Positive samples were amplified by conventional PCR and sequenced utilizing the Sanger method. The obtained sequences were aligned, and an evolutionary analysis was carried out using Bayesian inference. A total of 308 samples were tested. Of this total, 20 had a detectable viral load for Dengue, being detected DENV1 (18/20), co-infection DENV1 and DENV2 (1/20) and DENV4 (1/20). For Dengue serotype 3 and for the CHIKV, ZIKV, MAYV and OROV viruses, no individuals with a detectable viral load were found. A total of 9 of these samples were magnified by conventional PCR for sequencing. Of these, 6 were successfully sequenced and, according to the evolutionary profile, 5 corresponded to serotype DENV-1 genotype V, and 1 to serotype DENV-4 genotype II. In the study, we demonstrate co-circulation of the DENV-1 genotype V and the DENV-4 genotype II. Co-circulation of several DENV serotypes in the same city poses a risk to the population and is correlated with the increase of the most severe forms of the disease. Similarly, co-circulation of genetically distinct DENV and the occurrence of simultaneous infections can affect recombination events and lead to the emergence of more virulent isolates.

## 1. Introduction

The term arbovirus (*Arthropod-Borne Virus*) refers to a group of different virus families that are transmitted by arthropod vectors, such as mosquitoes, ticks and flies [1,2]. Although arboviruses develop a natural zoonotic cycle, involving the virus, its vectors, non-human vertebrate hosts and the environment [3], they are transmitted to humans accidentally or to other vertebrates during the bite by an infected vector. 

Most arboviruses that cause human disease belong to three families: *Togaviridae* (genus *Alphavirus*), *Flaviviridae* (genus *Flavivirus*) and *Bunyaviridae* (genera *Bunyavirus*, *Orthobunyavirus, Nairovirus* and *Phlebovirus*), but three other families also include these arboviruses: *Rhabdoviridae*, *Orthomyxoviridae* and *Reoviridae* [4,5]. A group among these stands out for harboring species that represent a worldwide public health problem, the *Flaviviruses:* DENV, West Nile Virus (WNV), Zika Virus (ZIKV) and Yellow Fever Virus (YFV), all with a worldwide distribution [3,6,7]. Species such as Japanese Encephalitis Virus (JEV), Tick-borne Encephalitis Virus (TBEV), Venezuelan Equine Encephalitis Virus (VEEV) and Encephalitis de St. Virus. SLEV) are generally restricted to specific regions [4,8]. The main vectors are the mosquitoes *Aedes aegypti* and *Aedes albopictus* [9,10,11,12,13].

With a predominantly tropical climate, Brazil is a country of high territorial dimensions, encompassing several biomes where the environment favors the development of some of the main arbovirus vectors and makes it a propitious place for the occurrence of arboviruses [8,14]. In Brazil’s northern region, the Amazon joins abundant natural biodiversity, which also includes a big variety of arboviruses. Over 150 species of arboviruses have already been cataloged in this region, predominantly in the states of Pará and Amazonas [15].

Considering the scenario, which includes an ample variety of species allied to the normal and extensive genetic variation of the viruses, the determination of types and genotypes of viral strains using phylogenetic analyses tools is considered an important tool for elucidating how the virus disseminates, from an evolutionary perspective [16]. This could help in resolving hypothetical outbreaks, investigating their etiology in a space/time context [17]. As a practical example of these applications, the first report of Zika virus transmission using vectors was observed in France in 2019. A few months after the ecological identification, it became possible by using certain tools to isolate the Asiatic general type of the circulating virus, which suggests that it originated in the Asian Southeast [18,19].

Another study, through phylogenetic reconstruction, suggested that the introduction of the CHIKV ECSA (East/Central/South African) genotype in Brazil occurred separately in several locations, as opposed to a single location, then followed by viral dissemination, as expected [20,21]. To highlight the importance of investigating these variants, the ECSA genotype (East/Central/South African), when compared to the African genotype S-27 (S-27) in mice, caused a high and acute fatality, with a higher viral load in the central nervous system and peripheral organs [22]. This genotype has endured in northeastern Brazil, since its first detection in the country in 2014 [23], which shows concern about its circulation and its possible consequences on the population’s health.

DENV also has peculiarities regarding its variants. The incidence and severity of the disease caused by it is related to circulating serotypes as well as to genotypes, which differ not only in genetic content but also in geographic distribution, where it is possible to observe that the introduction or reemergence of a serotype in a given region may be the triggering factor for disease outbreaks [24,25,26,27,28,29,30].

Located in the Brazilian Amazon region, with reports already describing the presence of some arboviruses being circulated [31,32,33,34], the state of Rondônia has high numbers for acute febrile illnesses, a common clinical symptom of arboviruses. Many of these are described as viruses, remaining without laboratory diagnostic confirmation and molecular characterization. In 2008, Vieira and collaborators detected 160 patients infected with DENV distributed in 7 municipalities in the state, including the capital Porto Velho [31].

The high numbers of Dengue fever in Porto Velho is a problem that has been observed for some time. Between 2000 and 2008, the number of cases was higher than the national average, in the North region and in the same state of Rondônia. It also concentrated most of the severe cases of the disease [35], and recently, Aedes aegypti mosquitoes were found infected by ZIKV and DENV, confirming local transmission [36].

In this context, the present study aimed to test the circulation of 5 arboviruses (DENV, CHIKV, ZIKV, Mayaro virus and Oropoche virus) and to perform phylogenetic and co-infection characterization to provide data towards understanding how the virus disseminates, how the disease evolves and then to enable the planning of prevention strategies.

## 2. Materials and Methods

### 2.1. Location of Study

The study was carried out at the Molecular Virology Laboratory of the Fundação Oswaldo Cruz (FIOCRUZ) and at the Centro de Medicina Tropical de Rondônia (CEPEM), in the city of Porto Velho, Rondônia, Brazil. 

### 2.2. Samples

Biological samples were collected between January 2018 and December 2019, at the Ambulatório de Malária do CEPEM. For this study, serum samples from 308 patients with a history of acute febrile illness were used, which tested negative for malaria (using microscopy) and with signs and symptoms suggestive of arboviruses (headache, myalgia, joint pain or rashes). These were evaluated by a health team that collected clinical, demographic and laboratory data. Individuals over 12 years of age, of both sexes and whose symptoms did not exceed five days were included. Patients without laboratory characterization, indigenous patients and those with uncontrolled co-morbidities were excluded.

### 2.3. Ethics Declaration

The study was carried out in accordance with the ethical principles stipulated by the 1975 World Medical Assembly and the Ministry of Health (Resolution 466). The project was evaluated and approved by the ethics committee of the Centro de Pesquisa em Medicina Tropical—CEPEM—Rondônia under brief No. 1474.102 in 1 April 2016.

### 2.4. Analyses

#### 2.4.1. RT-qPCR for Zika, Dengue and Chikungunya

Viral RNA was isolated from 140 µL of serum sample using the QIAamp Viral RNA Mini Kit (QIAGEN, Hilden Germany), according to the manufacturer’s instructions and eluted in 60 µL of AVE buffer. RNA extractions were tested for the presence of ZIKV viruses; DENV serotypes 1, 2, 3 and 4; and CHIKV for the real-time PCR kit, Kit Biomol ZDC (Instituto de Biologia Molecular do Paraná, Brazil), following the manufacturer’s instructions. The reactions were performed on a Real-Time 7500 PCR System from Applied Biosystems, with the reaction profile: 51 °C for 30 min, 95 °C for 15 min, 40 cycles of 95 °C for 15 s and 60 °C for 1 min. The result was considered positive when the cycle threshold was ≤36 for the analyzed virus and ≤28 for the internal control.

#### 2.4.2. RT-qPCR for the Mayaro (MAYV) and Oropouche (OROV) Viruses

To detect MAYV and OROV viruses, 2.5 µL of extracted RNA was incubated in a final volume of 10 µL of reaction containing 5 µL of Gotaq Mix 2×, 0.2 µL of GoScript 50×, 100 nM of MAYV probe, 100 nM of OROV probe, 300 nM of Primer Mix MAYV and 300 nM of Primer Mix OROV. The testing was performed on a Real-Time 7500 PCR System from Applied Biosystems, with the reaction profile: 45 °C for 15 min, 95 °C for 15 min, 45 cycles of 95 °C for 15 s and 60 °C for 1 min. The result was considered positive when the cycle threshold was less than 38 [37].

#### 2.4.3. Reverse Transcription

Complementary DNA (cDNA) was produced starting from the RNA extracted from positive RT-qPCR samples, using the Reverse Transcription (RT) method using the enzyme M-MLV Reverse Transcriptase (© 2020–2011, 2016 Promega Corporation, Madison, USA). Extracted total RNA (2 µg) was incubated with 0.5 µg of random primer, at a temperature of 70 °C for 5 min in order to undo the secondary structures. Subsequently, 5 µL of the 5× M-MLV Reaction Buffer, 5 µL of 10 mM dNTP Mix, 25 units of RNAse Inhibitor, 200 units of M-MLV RT, in a final volume of 25 µL, were incubated for 60 min at 37 °C.

#### 2.4.4. PCR for DENV Sequencing

The cDNA was amplified with conventional PCR in a volume of 50 uL, using the previously described D1 and D2 *primers* (Table 1) to amplify a PCR product 511 Base Pairs (bp) [38]. The reaction was adapted according to the following conditions: 20 mM Tris HCl (pH 8.4), 50 mM KCl, 1.5 mM MgCl_2_, 200 µM of each Triphosphate Deoxynucleotide (dNTP), 200 nM Primer D1, 200 nM Primer D2 and 6 Units of Taq DNA Polymerase Hot Master enzyme. After adding 10 µL cDNA, the reaction was incubated in the thermal cycler at 94 °C for 3 min for initial denaturation, followed by 35 cycles at 94 °C for 30 s, 55 °C for 1 min for annealing *primers* and extension at 72 °C for 2 min, ending at 72 °C for 5 min. The PCR product was visualized using the agarose gel electrophoresis method.

The conventional PCR product was purified using the enzyme ExoSAP (Cellco) as instructed by the manufacturer and sequenced by the automated Sanger method, carried out in partnership with the DNA Sequencing Technological Platform (RPT01B IGM) of the Fundação Oswaldo Cruz Bahia—FIOCRUZ/BA. The consensus sequences were produced manually using the MEGA7—Molecular Evolutionary Genetic Analysis software [39], having as evaluation basis for this procedure the Phred note of the electropherogram, where priority was given to bases whose values were greater than or equal to 30 (1 probability of error in 1000) [40].

#### 2.4.5. Evolutionary Analysis 

Through systematic research with the descriptor *“DENV- (serotype number) isolate complete genome*” in Genbank (available at: https://www.ncbi.nlm.nih.gov/nucleotide/), currently associated with the National Center for Biotechnology Information (NCBI), as well as the papers [41,42], two banks of sequences to elucidate the evolutionary lineage of the isolated strains were assembled based on the purpose of the analyzes: (1) Determination of serotypes and (2) Genotyping of serotypes. Sequences with information about the collection year and isolation location were prioritized. The sample sequences were aligned with each bank of sequences under the MUSCLE algorithm in the MEGA7 software. The alignment was cut to remain only the nucleotides belonging to the region of the samples that had been amplified, removing the remaining bases that were present in the bank sequences.

The evolutionary model Tamura-Nei with Gamma distribution and Invariant Sites (TN93 + G + I) was chosen using the tool attached to MEGA7 Model Analysis. The evolutionary analysis was performed with Bayesian inference using Monte Carlo Markov Chain (MCMC) algorithms implemented in the BEAST v.1.10.4 package. [43]. The evolutionary profile was estimated using the Bayesian Skyline coalescent model, relaxed molecular clock not correlated with the lognormal distribution and under a chain length of 1 × 10^8^, with a collection at every 10,000. The analyzes were performed in duplicate and were combined when using the LogCombiner software v.1.8.4. to evaluate the run in the Tracer software v.1.7.1, which showed good convergence of the parameters (Effective Sample Size—ESS value> 200). The TreeAnnotator v.10.1 and FigTree v1.4.3 programs were used to summarize the later distribution of the tree with a 10% burn-in and to view/customize the annotated MCC (Maximum Clade Credibility) tree, respectively.

#### 2.4.6. Statistical Analysis

Statistical analysis was performed using the estimated p-value using the Chi-square test and test *t* ANOVA for the frequencies of the viral agent and the symptoms presented using IBM SPSS Statistics 20 software.

## 3. Results and Discussion

A total of 308 individuals were included in the study during the years 2018 and 2019, following established inclusion and exclusion criteria, selected for convenience, representing approximately 2.24% of 13,758 patients where there was an initial suspicion of malaria. Serum samples were collected from each participant, as well as the clinical data needed for the study. All 308 patients had a negative result in the *Plasmodium sp*. 65.6% (202/308) of the patients were male and 34.4% (106/308) were female. All individuals seen had an acute febrile condition with onset of symptoms ranging from 1 to 5 days prior to the date of the consult. 

The RNA extracted from the biological samples of the 308 patients were tested in Real Time PCR assays. Of this total, 20 samples had a detectable viral load for Dengue, which demonstrated a prevalence of 6.5%, with the DENV1 (18/20), co-infection of DENV1 and DENV2 (1/20), and DENV4 (1/20) viruses being detected. For Dengue serotype 3 and for the CHIKV, ZIKV, MAYV and OROV viruses, no individuals with a detectable viral load were found. For comparison purposes, in 2019 the incidence of dengue in Porto Velho, based on notified and confirmed cases, was 0.09% and 0.03%, respectively. A confirmed to notified ratio of 29.90% was reported (data provided by Health Department of the city of Porto Velho). These data indicate that the sample selection based on an initial hypothesis of dengue or arbovirus in health care facilities is more sensitive when compared to patients in the malaria outpatient clinic. In addition, the serological method used for confirmatory diagnosis by the SEMUSA of Porto Velho allows a longer interval for recruiting participants when compared to the molecular method. With a short fever onset interval, inflammatory processes may not have been localized, resulting in a non-specific diagnostic suspicion.

**Figure 1:**
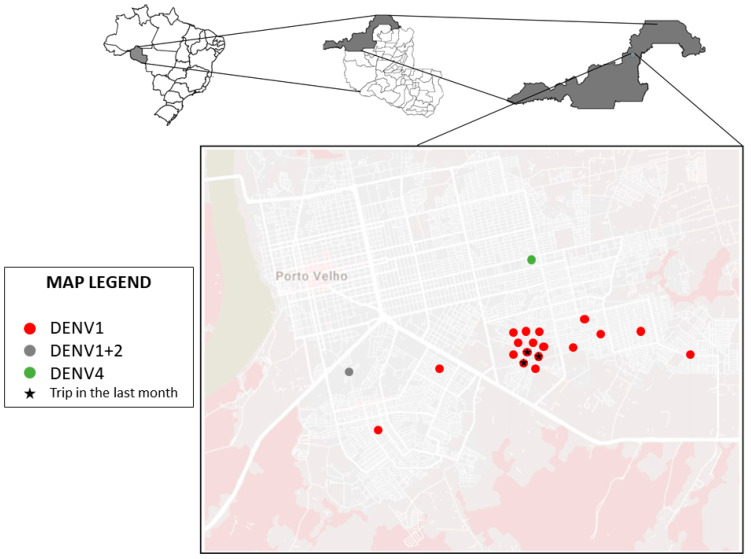
Distribution map of dengue cases identified in the study.

The geographic distribution of the isolated cases is shown in Figure 1, which allows for observing the proximity of the neighborhoods where the infections were detected. All individuals were residents of the city of Porto Velho, Rondônia, and only 3 reported traveling within 30 days prior to the consult to a district in the municipality of Porto Velho.

All samples were collected in the east and south neighborhoods of the municipality of Porto Velho, a region relatively removed from the city center and with poorer basic sanitation services. In 2019, the Associação Brasileira de Engenharia Sanitária e Ambiental (ABES), prepared a ranking on the universal availability of sanitation in Brazil. This document detailed how the farther away a location is from the country’s south-southwest, the worse the sanitation conditions are. The city of Porto Velho was considered the worst capital in the country regarding universal sanitation, where only 4.58% of the population has access to sewage collection and only 3.19% has access to the treatment of this waste [44]. Factors such as these may be too closely associated with causing DENV circulation in the region [45,46]. This local transmission was confirmed in entomological-virologic survey sampled mosquitoes (Diptera: Culicidae) in outdoor and indoor from dwellings at Porto Velho, state of Rondônia where Aedes aegypti was found infected by ZIKV (one pool of three females sampled in 2016) and DENV-4 (one male sampled in 2018) [36].

Among the signs and symptoms reported by viremic patients during anamnesis and physical examination (Table 2), there were 20/20 fever (100%), 19/20 chills (95%), 18/20 muscle pain (90%), headache 17/20 (85%), joint pain 15/20 (75%), nausea 15/20 (75%) and retro-orbital pain 14/20 (70%). This type of clinical manifestation is common in infectious processes that trigger an exacerbated inflammatory response, and although non-specific, it is considered characteristic in dengue without alarm signals, with additional symptoms such as vomiting and the appearance of *rash*, around 3 to 4 days after the onset of fever [47]. Some patients had alarm signals, such as 6/20 vomiting (30%) and 6/20 abdominal pain (30%) [48,49], in addition to 25% (5/20) having a history of DENV infection, which can be a predictor of a severe case of the disease if there are successive infections by different serotypes [50]. The study was not designed to compare between groups, although an exploratory analysis was conducted, and the only statistical significative result was lack of appetite. It only aids in describing the selected sample. As for pre-existing conditions, diabetes (1/20) and hypertension (1/20) were reported. Only 2 individuals claimed to use medication to treat fever symptoms. None of the individuals had prolonged use of any medication, or illicit drugs, and 45% (9/20) had a history of malaria.

While the research was ongoing, the epidemiological bulletin of the Secretaria de Vigilância em Saúde (Health Ministry) recorded 994 probable cases of Dengue fever between the years 2018 (419) and 2019 (575), with a 37.2% increase in the number cases for these years. According to the same document, there were no deaths from Dengue in the state [51]. Despite the number of probable cases in 2018 and 2019 for Chikungunya (155) and Zika (67), they were not detected in the population study [51].

In addition to the high prevalence, the co-circulation of DENV serotypes 1, 2 and 4 detected in this study highlights the endemicity of the disease in the region and the need for a molecular diagnosis to characterize the serotypes, since this factor is of concern. Secondary infections by serotypes other than the primary infection may trigger more severe clinical conditions, due to the potentiation of the infection by the action of pre-existing non-neutralizing heterologous antibodies in the patients’ serum [25].

This scenario of simultaneous circulation was also reported by Bastos and collaborators in 2011, involving the 4 dengue serotypes in the city of Manaus, Amazonas, which is a neighboring state of Rondônia [26]. Despite non-detection of the DENV serotype 3, its circulation in the state of Rondônia and in the city of Porto Velho has already been reported in literature, highlighting that there is a possibility of this same scenario, as observed in the state of Amazonas, being repeated in Rondônia [31].

**Figure 2:**
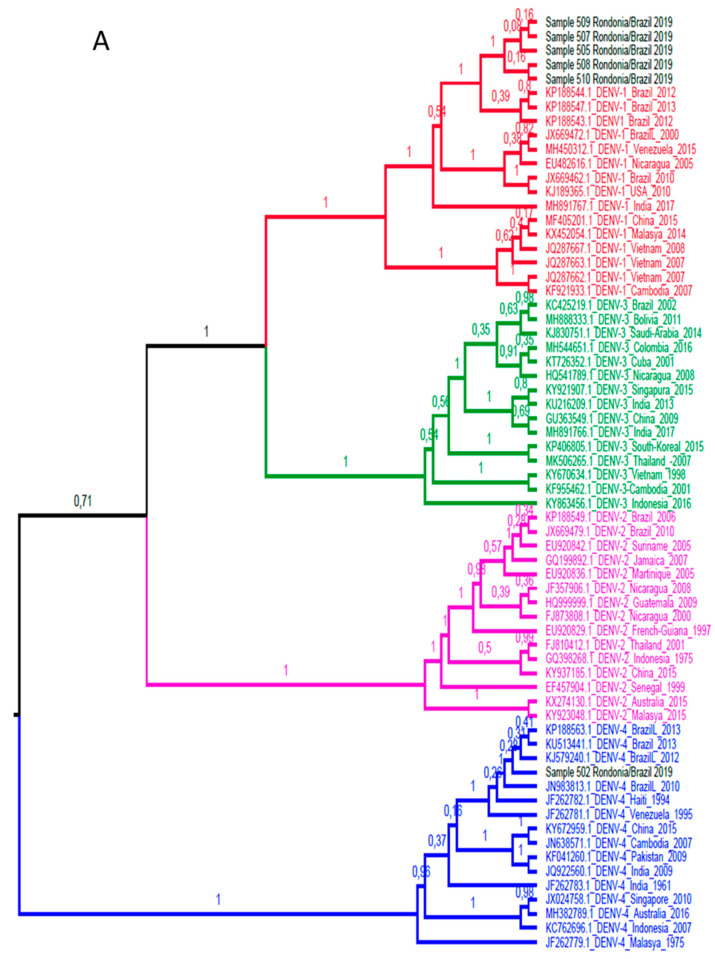

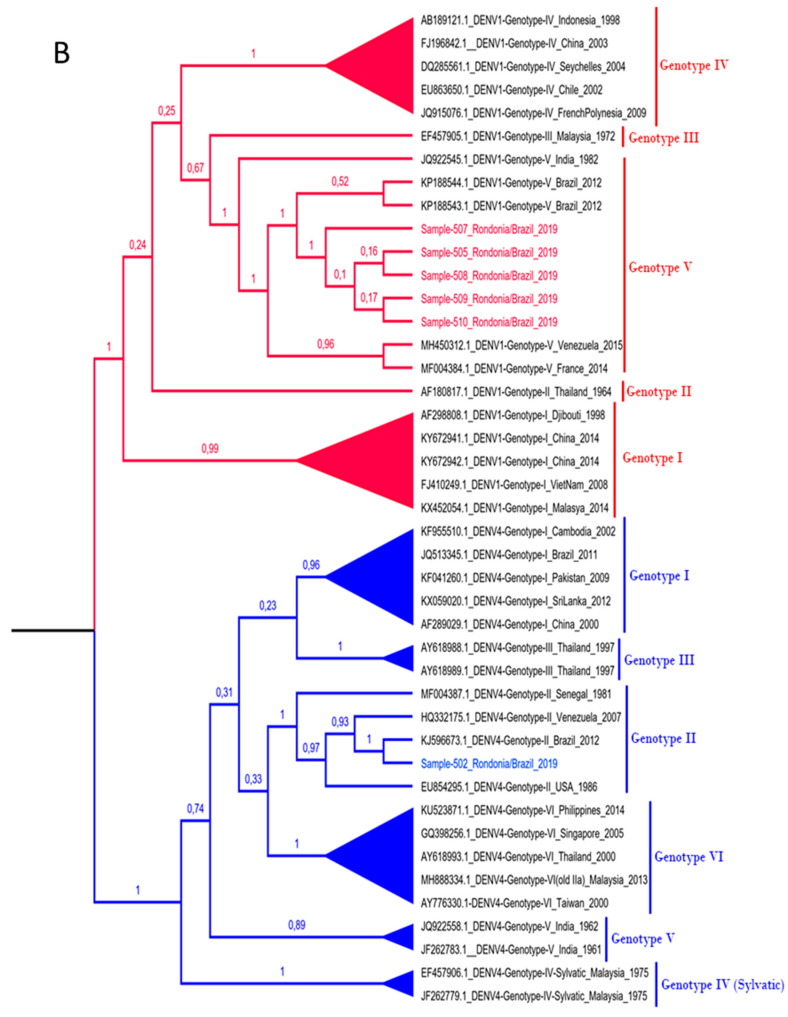
(**A**). Bayesian phylogenetic tree for determination of serotypes. The tree of maximum clade credibility generated. Fifteen sequences of each DENV serotype were used for this evolutionary lineage determination, totaling 60 sequences. The access codes are shown in the identification of the taxa. The clades are colored according to the DENV serotype (red, DENV-1; pink, DENV-2; green, DENV-3 and blue, DENV-4). The samples correspond to the black colored taxa. The posterior support in decimal format is shown in each node. (**B**). Bayesian phylogenetic tree for determination of genotypes. The tree of maximum clade credibility generated. A total of 37 DENV sequences were used for this evolutionary lineage determination, distributed in DENV-1 (17 sequences) and DENV-4 (20 sequences) genotypes. The access codes are shown in the identification of the taxa. The clades are colored according to the DENV serotype, being red for DENV-1 and blue for DENV-4. Sample sequences are colored according to the serotype to which they belong. The identification of the genotypes is displayed in each taxon, as well as in the colored vertical bars to the right of the taxa. The posterior support in decimal format is shown in each node.

Among the 20 dengue samples with detectable viral load, due to the sensitivity of the method, only 9 were amplified by conventional PCR for sequencing. A total of 6 samples were successful in sequencing the *sense* and *antisense* tapes and according to the evolutionary profile elucidated by the Bayesian analysis (Figure 2), with 100% posterior supporting reliability, 5 samples corresponded to strains belonging to the DENV-1 serotype, and only 1 to the DENV-4 serotype. All strains detected evolved with isolated strains in South American countries, demonstrating even greater proximity to isolated specimens in Brazil, showing that the circulation of DENV in the region remains closed off and restricted to the country, with no introduction detected of virus specimens from other countries in the region’s DENV urban cycle.

Another factor that supports this consideration is the formation of a monophyletic group with 100% support in the subsequent probability of the DENV-1 sample sequences. This phylogenetic profile is different from the findings of Vieira et al. during the 2001 to 2003 epidemics in Porto Velho, where the serotype 1 of the isolated dengue virus showed a 98% similarity with isolated sequences in Rio de Janeiro, suggesting that the virus that caused epidemics at that time was derived from the southern regions of the country [33].

On the other hand, the prevalence of serotypes in Porto Velho also differed in relation to the Brazilian case. Until 2016, the prevalence of dengue serotype 1 in Brazil was higher than serotype 2, being 97.24% and 0.69%, respectively [52]. As of 2017, this situation has been dramatically reversed, where the number of DENV-2 cases increased significantly across the country, it being the most prevalent serotype (54.3%) [53]. Despite the national context, serotype 1 was the most isolated of the population study, being responsible for 96.4% of the cases detected. The detection of DENV-2 cautions us about the onset of severe illnesses, since in the last 20 years DENV-2 activity in Brazil has contributed significantly to changes in disease morbidity [27], as it is related to the development of severe cases of dengue fever [28,54,55,56].

Only one case of co-infection was detected, involving serotypes 1 and 2. The patient in question did not show signs of alarm, reporting only the classic symptoms of the disease (headache, fever, muscle pain, rash). In the study by Dhanoa and colleagues, out of a total of 262 patients tested positive for DENV, 15.3% (40/262) were co-infections. Among the 40 co-infections detected, DENV-1/DENV-2 was identified in 85% of cases. The same study showed that the presence of alarm signs and severe manifestations of the disease was highly significant in co-infected patients, compared to mono-infected ones [57].

Despite the low rate of detection of DENV-4, several studies correlate the events of the reintroduction and reemergence of this serotype with epidemic outbreaks of the disease. DENV-4 was first identified in Brazil in 1982, in the state of Roraima [58]. In the state of Amazonas, the first detection occurred between 2005 and 2007, being the first case reported in Brazil after 25 years [59]. In 2012 and 2013, the emergence of DENV-4 combined with a highly susceptible population, and previously immunized by DENV-1, gave rise to the most severe epidemic observed in the state of Minas Gerais, Brazil [29]. In 2014, the same situation was observed in Mato Grosso do Sul, Brazil, with the introduction of DENV-4 also triggering an epidemic outbreak [30]. Although there are no studies describing the events of the introduction of DENV-4 in the state of Rondônia, the detection of this serotype highlights its endemicity and the need for epidemiological and molecular studies in the region, since the detection of the serotype can be an indication of reintroduction and reemergence. 

In the study, we show the existence of DENV-1 genotype V and DENV-4 genotype II co-circulation. The current diversity of DENV-1 in the Americas resulted from two independent introductions of the V genotype from India around the early 1970s and 1980s [60]. The two strains of genotype V were probably introduced to the Americas through the Caribbean and later disseminated on the continent, confirming the central role of the Caribbean islands in the initial dispersion of DENV from Asia to the Americas [61,62,63,64]. The DENV-4 Genotype II is widely distributed with strains from all parts of Southeast Asia (Indonesia, Malaysia, Singapore), China, Western Pacific Ocean Islands, Australia, Caribbean and the Americas [65,66]. Co-circulation of several DENV serotypes in the same city poses a risk to the population and is correlated with the increase of the most severe forms of the disease [67,68,69]. Similarly, co-circulation of genetically distinct DENV, even at the genotype level, and the occurrence of simultaneous infections can affect recombination events and lead to the emergence of more virulent isolates [41,67].

## 4. Conclusions

The findings confirm the co-circulation of two genotypically distinct serotypes of DENV in the state of Rondônia, with internal circulation, without the detection of viral specimens from other countries, which highlights the need for prevention strategies, due to the possibility of there being grave cases of the disease.

## Figures and Tables

**Table 1:** DENV 1–4 Conventional PCR primers.

Primer	Sequence (5’–3’)	Position	DNA Size (pb)
D1	TCAATATGCTGAAACGCGCGAGAAACCG	134–161	511 pb
D2	TTGCACCAACAGTCAATGTCTTCAGGTTC	616–644	

Adapted from LANCIOTTI et al. 1992 [38].

**Table 2:** Signs and symptoms of individuals tested positive.

Signs and Symptoms	Negative DENV		Positive DENV				
	N = 288	%	N = 20	%	Chi-Square	*p* Value	Test t ANOVA
CEFALEIA	260	90.3%	17	85.0%	1.495	0.474	0.290
FEVER	258	89.6%	20	100.0%	1.619	0.203	0.205
CHILLS	243	84.4%	19	95.0%	1.139	0.286	0.288
SWEATING	175	60.8%	12	60.0%	0.069	0.792	0.793
NAUSEA	157	54.5%	15	75.0%	2.244	0.134	0.135
VOMIT	88	30.6%	6	30.0%	0.140	0.708	0.710
MUSCLE PAIN	255	88.5%	18	90.0%	0.000	0.988	0.988
JOINT PAIN	234	81.3%	15	75.0%	0.894	0.344	0.346
DIARRHEA	78	27.1%	3	15.0%	2.006	0.157	0.158
DYSURIA	47	16.3%	4	20.0%	0.027	0.869	0.869
OLIGURIA	64	22.2%	2	10.0%	2.248	0.134	0.135
LACK OF APPETITE	169	58.7%	11	55.0%	15.563	0.001	0.729
RASH	18	6.3%	2	10.0%	0.201	0.654	0.655
STAINS	24	8.3%	2	10.0%	0.003	0.953	0.954
PRURITUS	20	6.9%	1	5.0%	0.245	0.621	0.622
DIZZINESS	140	48.6%	11	55.0%	0.074	0.786	0.787
VERTIGO	86	29.9%	8	40.0%	0.384	0.536	0.537
MENTAL CONFUSION	29	10.1%	1	5.0%	0.843	0.359	0.360
DYSPNEA	62	21.5%	5	25.0%	0.027	0.870	0.871
BLEEDING	8	2.8%	0	0.0%	0.676	0.411	0.413
ABDOMINAL PAIN	133	46.2%	6	30.0%	2.767	0.096	0.097
RETRO-ORBITARY PAIN	174	60.4%	14	70.0%	0.722	0.697	0.428
WEIGHT LOSS	129	44.8%	1	5.0%	0.310	1.400	0.238
